# Effect of integrin receptor-targeted liposomal paclitaxel for hepatocellular carcinoma targeting and therapy

**DOI:** 10.3892/ol.2021.12611

**Published:** 2021-03-03

**Authors:** Liyu Chen, Yanbin Liu, Weiya Wang, Kai Liu

Oncol Lett 10: 77-84, 2015; DOI: 10.3892/ol.2015.3242

Subsequently to the publication of the above paper, it has been brought to the attention of the Editor that there were a number of striking similarities between the article and another paper published in a different journal by different authors located at different research institutions. First, numerous textual similarities were noted between several paragraphs of the text comparing between this paper and the other paper, which was published in *Oncology Reports*, also in 2015: Wang R-H, Cao H-M, Tian Z-J, Jin B, Wang Q, Ma H and Wu J: Efficacy of dual-functional liposomes containing paclitaxel for treatment of lung cancer. Oncol Rep 33: 783–791, 2015. Furthermore, Fig. 9 in the above article contained data plots that were also strikingly similar to those included in Fig. 9 in the paper published in *Oncology Reports*.

After having considered these aspects, the Editor of *Oncology Letters* has decided that the above paper should be retracted from the publication on the grounds of a lack of certainty regarding the independence and integrity of the study. The authors were contacted in this regard, but did not offer any rebuttal in light of the Editor's decision to retract this paper. Note that the study by Wang *et al* in *Oncology Reports* has also been retracted. The Editor regrets any inconvenience that this retraction has caused.

